# A New Chemical Pathway Yielding A-Type Vitisins in Red Wines

**DOI:** 10.3390/ijms18040762

**Published:** 2017-04-04

**Authors:** Paula Araújo, Ana Fernandes, Victor de Freitas, Joana Oliveira

**Affiliations:** Laboratório Associado para a Química Verde, Departamento de Química e Bioquímica, Faculdade de Ciências da Universidade do Porto, Rua do Campo Alegre, 687, 4169-007 Porto, Portugal; paula.araujo@fc.up.pt (P.A.); ana.fernandes@fc.up.pt (A.F.); vfreitas@fc.up.pt (V.F.)

**Keywords:** malvidin-3-*O*-glucoside, pyruvic acid, oxaloacetic acid, carboxypyranomalvidin-3-*O*-glucoside vitisin, anthocyanins, red wines

## Abstract

A new chemical pathway yielding A-type vitisins in red wines is proposed herein from the reaction between anthocyanins and oxaloacetic acid (OAA). This new chemical path is thought to occur in the first stages of the wine production even during the fermentation process. This is due to the revealed high reactivity of OAA with anthocyanins compared with the already known precursor (pyruvic acid, PA). In model solutions at wine pH (3.5), when malvidin-3-*O*-glucoside (mv-3-glc) is in contact with OAA and PA a decrease in the OAA concentration is observed along with the formation of A-type vitisin. Moreover, part of the OAA is also chemically converted into PA in model solutions. The reaction yields were also determined for OAA and PA using different mv-3-glc:organic acid molar ratios (1:0.5, 1:1, 1:5, 1:10; 1:50, and 1:100) and these values were always higher for OAA when compared to PA, even at the lowest molar ratio (1:0.5). The reaction yields were higher at pH 2.6 in comparison to pH 1.5 and 3.5, being less affected at pH 3.5 for OAA. These results support the idea that OAA can be at the origin of A-type vitisins in the first stages of wine production and PA in the subsequent ageing process.

## 1. Introduction

Color is an important characteristic that is directly associated with the quality of red wines. Anthocyanins, the compounds responsible for this color, are flavonoid pigments formed in berries from *verasion* onwards via the phenylpropanoid pathway [1]. These pigments are slowly extracted from grape skins during maceration/fermentation into the must and wine. At this stage, diverse anthocyanin-derived pigments, namely pyranoanthocyanins and especially those derived from malvidin-3-*O*-glucoside (the most abundant anthocyanin in grapes), are formed by its condensation with the metabolites released by yeasts during fermentation [2,3,4,5].

A-type vitisins are one of the main pyranoanthocyanins found in red wines during ageing [6] and are described in the literature to be formed from the reaction of anthocyanins with pyruvic acid (PA) released into the must by yeasts during alcoholic fermentation [3,7,8,9,10]. Malolactic fermentation can also influence the concentration of this organic acid in wines [11].

The importance of these anthocyanin-derivatives to the overall red wine color arises from their chromatic stability in aqueous solution, the absence of hydration reactions [12], and their increased stability towards bleaching by SO_2_ when compared to the original anthocyanins [13]. Moreover, A-type vitisins have already been described to be the precursors of other pyranoanthocyanins in red wines, namely A and B-type portisins, pyranoanthocyanin dimers, and oxovitisins [14,15,16,17].

Oxaloacetic acid (OAA) is an organic acid produced during fermentation from the activated enolic form of PA (phosphoenol pyruvate) that adds a nucleophile to the carbon dioxide [18]. OAA is also a precursor by transamination of aspartic acid in wines [18]. Moreover, in grapes, aspartic acid constitutes a reserve of OAA which can be transformed into malic acid or into sugar, depending on the demand during the maturation [19]. Although the levels of OAA in musts and wines are expected to be low, its quantification in those matrices is not well documented in the literature [20]. This is due to the lack of a reliable and sensitive method for the simultaneous detection of various keto-acids and due to the instability of OAA in severe reaction conditions [21].

**Figure 1:**
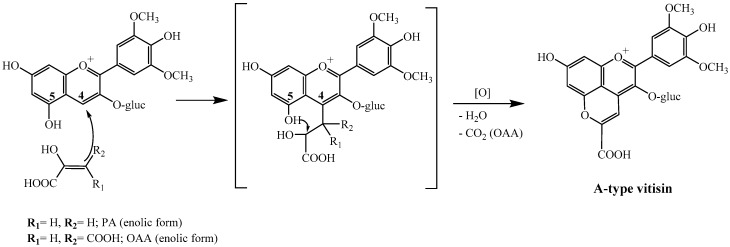
Mechanism of formation of A-type vitisin (carboxypyranomalvidin-3-*O*-glucoside) from pyruvic acid (PA) and oxaloacetic acid (OAA).

In this work, a new chemical pathway yielding A-type vitisins (Figure 1) in red wines is proposed from the reaction of anthocyanins with OAA. The detection and quantification of this organic acid in white and red grape must samples in different points of the fermentation process is also described.

## 2. Results and Discussion

### 2.1. Reactivity of Anthocyanins with Oxaloacetic Acid (OAA) and Pyruvic Acid (PA)

**Figure 2:**
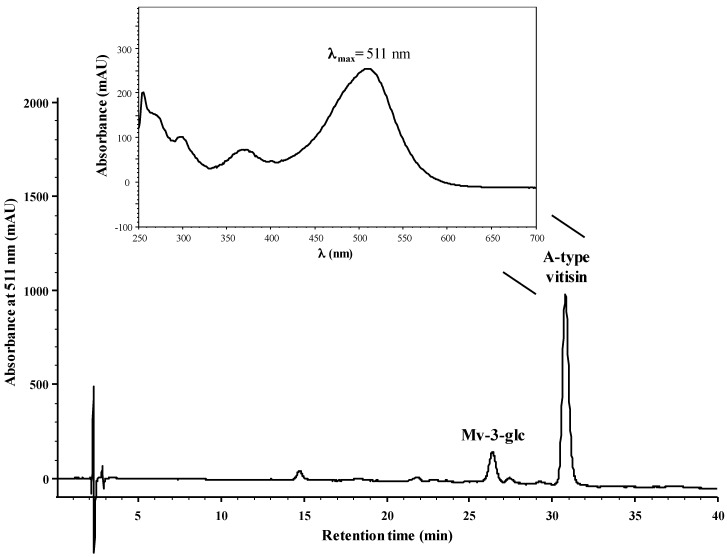
HPLC chromatogram recorded at 511 nm of a model solution containing mv-3-glc and OAA at pH 3.5 after 1 day of reaction at room temperature. Insert: UV-Visible spectrum and maximum absorption wavelength (511 nm) of the A-type vitisin recorded from the HPLC diode array detector.

The reactivity of mv-3-glc with OAA was evaluated in model solution at wine pH (~3.5). After 1 day of reaction at room temperature it was possible to detect by HPLC-DAD the appearance of a new chromatographic peak (Figure 2) presenting a UV-Visible spectrum (λ_max_ = 511 nm) (see Figure 2) similar to the one already observed for A-type vitisin. The identity of the peak was confirmed by LC-MS in the positive ion mode, revealing the ion mass [M]^+^ at *m*/*z* 561 and the fragment at *m*/*z* 399 (loss of a glucose moiety), which agrees with the data reported for A-type vitisin (see Figure S1).

The mechanism of formation of these pigments from OAA in red wines is expected to be similar to the one reported in the literature for PA [13,22], with the exception that in the case of OAA a decarboxylation is observed in the final step of the reaction (Figure 1), similar to what occurs with acetoacetic acid [5,22].

**Figure 3:**
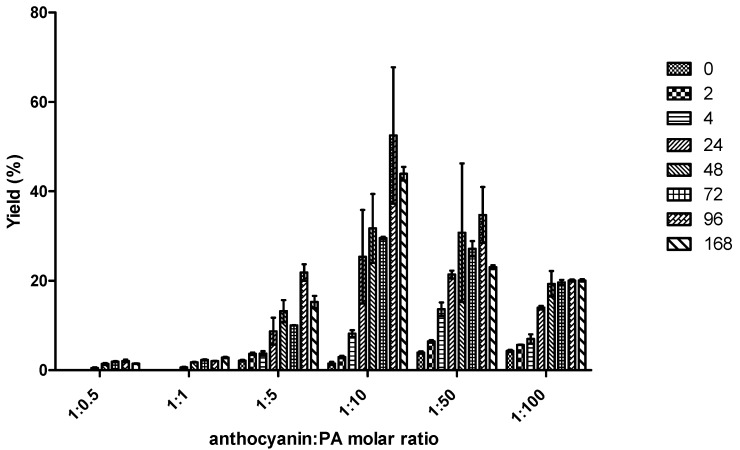
Mean of the yields (%) (triplicate) ± SD of formation of A-type vitisin after 0, 2, 4, 7, 24, 48, 72, 96, and 168 h of reaction between mv-3-glc and PA at different molar ratios mv-3-glc:PA 1:0.5, 1:1, 1:5, 1:10, 1:50, and 1:100 at pH 2.6 and room temperature.

**Figure 4:**
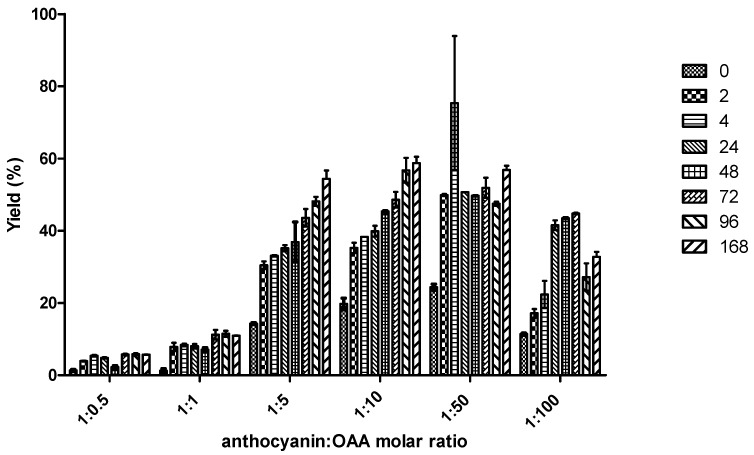
Mean of the yields (%) (triplicate) ± SD of formation of A-type vitisin after 0, 2, 4, 24, 48, 72, 96, and 168 h of reaction between mv-3-glc and OAA at different molar ratios mv-3-glc:OAA 1:0.5, 1:1, 1:5, 1:10, 1:50, and 1:100 at pH 2.6 and room temperature.

To better understand the chemical pathways that drive the formation of A-type vitisins in red wines, the reactivity of anthocyanins with OAA and PA were evaluated independently in model solutions using different pH values (1.5, 2.6, and 3.5) and different anthocyanin:organic acid molar ratios from 1:0.5 to 1:100 during 7 days. In this study, mv-3-glc was used as an anthocyanin model. The results obtained are presented in Table 1 and Figure 3 and Figure 4.

Firstly, the reaction of mv-3-glc with PA or OAA was tested at three different pH values (1.5, 2.6, and 3.5) using a molar ratio of mv-3-glc:organic acid of 1:10. From the analysis of Table 1, it is possible to observe that the reactions yields were higher (statistically different for *p* < 0.05) at pH 2.6 when compared to pH 1.5 and 3.5 for both organic acids (OAA and PA). On the other hand, the reactivity of OAA with anthocyanins was higher than the one observed with PA (statistically different for *p* < 0.05) for all the pH values tested. Moreover, it was also possible to notice that the pH increase (from pH 2.6 to 3.5) affected the reaction yield of PA more notably than for OAA.

Using pH 2.6, six different molar ratios anthocyanin:organic acid (1:0.5, 1:1, 1:5, 1:10, 1:50, and 1:100) were tested for OAA and PA. In Figure 3 it is possible to observe that the best yield (53%) for the production of A-type vitisin from PA was obtained after 96 h for the molar ratio 1:10. On the other hand, in the case of OAA (Figure 4), the molar ratios do not seem to significantly influence the reaction yields. For instance, the maximum reaction yields obtained after 168 h for molar ratios 1:5, 1:10, and 1:50 were 54%, 59%, and 57%, respectively, which are statistically similar for *p* > 0.05.

Furthermore, the highest amounts of A-type vitisin were obtained sooner with OAA in comparison to PA. For instance, at molar ratio mv-3-glc:organic acid of 1:10, it can be verified that the formation yield of A-type vitisin in the case of OAA reached 38% after only 2 h of reaction (Figure 4) in comparison to the reaction with PA that yielded 3% (Figure 3). Although the maximum formation yields of A-type vitisin were not that different between both acids, the kinetic of formation from the OAA is faster than the one observed with PA. On the other hand, it is important to notice that the rate of formation of A-type vitisin from OAA decreases from 2 h until 168 h. This effect could be due to the chemical degradation of OAA into PA, which could further react with mv-3-glc to form A-type vitisin at a slower rate. Indeed, OAA is unstable in solution and is easily decomposed into PA by a decarboxylation reaction in model solution at pH 3.5 (see Figure S2).

**Figure 5:**
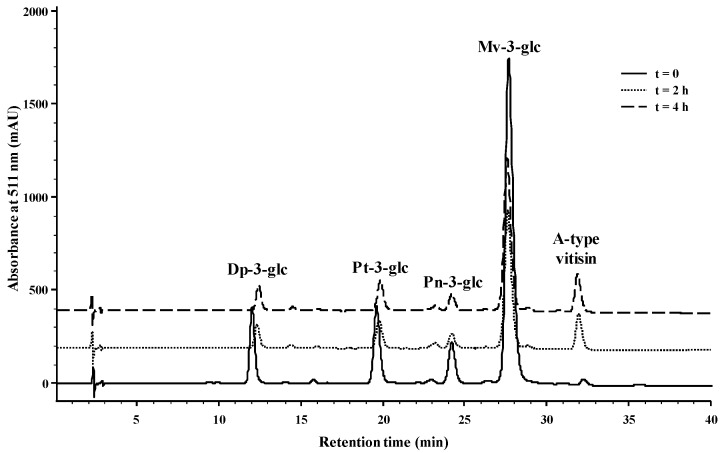
HPLC chromatograms recorded at 511 nm of a model solution (pH 3.5) containing the wine anthocyanins 3-monoglucoside dp-3-glc, pt-3-glc, pn-3-glc, and mv-3-glc (1.9 mM), PA (9.5 mM), and OAA (9.5 mM) at pH 3.5 at time 0, 2, and 4 h at room temperature.

**Figure 6:**
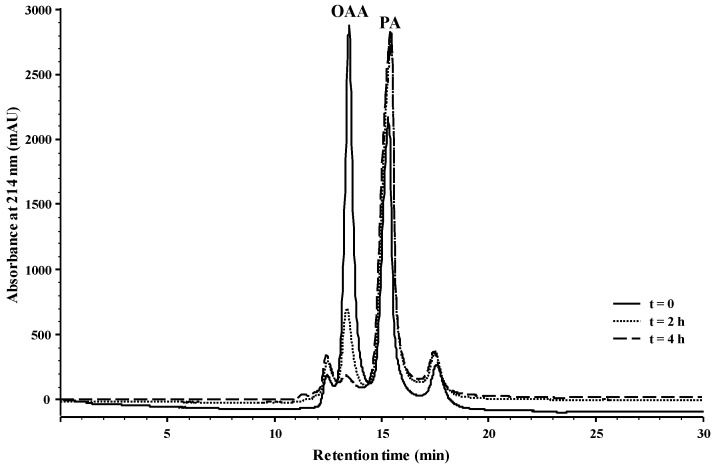
HPLC chromatograms recorded at 214 nm of a model solution (pH 3.5) containing the wine anthocyanins 3-monoglucoside dp-3-glc, pt-3-glc, pn-3-glc, and mv-3-glc (1.9 mM), PA (9.5 mM), and OAA (9.5 mM) at pH 3.5 at time 0, 2, and 4 h at room temperature.

Bearing all this in mind, it seems that the reactivity of OAA with anthocyanins is higher than the one observed for PA, since when both organic acids react individually with mv-3-glc, the yields of reaction are greater for OAA. To strengthen this idea, an experiment at wine pH (~3.5) was performed in which a mixture of anthocyanins-3-glc (1.9 mM) from wine was left to react with PA (9.5 mM) and OAA (9.5 mM) at the same time. In this experiment, two features were evaluated, the formation of the A-type vitisin from Mv-3-glc and the decrease of OAA and PA concentrations with time. In Figure 5, the HPLC chromatograms recorded at 511 nm show the decrease in the anthocyanin-3-glc, especially the more abundant mv-3-glc concentration and the increase of the respective A-type vitisin after 4 h of reaction at room temperature. The detection of OAA and PA in the same samples is presented in Figure 6, and the chromatograms showed the disappearance of OAA in 4 h concomitantly with the increase of PA. The decrease of OAA was due to the rapid reaction of this organic acid with the anthocyanin-3-glc to yield the A-type vitisin (as presented in Figure 3), but also owed to some chemical decomposition of this organic acid into PA, as demonstrated by the increase of the peak corresponding to PA in Figure 6 and in Figure 2 from the Figure S2.

Overall, a new chemical pathway is proposed leading to the formation of A-type vitisins in red wines from the reaction of anthocyanins with OAA. This new chemical pathway could be at the origin of the A-type vitisins found to occur in the first stages of wine production, even during the fermentation process, due to its increased reactivity with anthocyanins.

Thus, it is important to know the concentration of OAA and PA in grape musts and wines.

### 2.2. Detection and Quantification of OAA and PA in Grape Must

The concentration of OAA in grape must and wines is not well documented in the literature [20]. With this in mind, the levels of OAA and PA were determined in two grapes must samples (white and red) collected at different points of the alcoholic fermentation process (before, middle, and end). For this quantification, a *O*-(2,3,4,5,6-pentafluorobenzyl)oxime (*O*-PFBO) derivatization method was used (see S3 in Supplementary materials) coupled with Liquid Chromatography/Electron Spray Ionization-Mass Spectrometry (LC/ESI-MS) detection, which is selective for molecules comprising a keto group like keto-acids and presents low detection and quantification limits [21]. The concentration (Mean ± SD) of OAA and PA (in triplicates) obtained for the grape must samples are presented in Table 2.

According to the results showed in Table 2, grape must samples presented a higher concentration of PA in comparison to OAA. The content of PA increased along with the decrease in the sugar concentration until half of the sugar was consumed (R-MF and W-MF), and then stabilized (in the case of the red grape must) or decreased (white grape must). Usually, the concentration of PA increases until half of the sugar is consumed and then decreases up to the end of fermentation [23]. The different behavior observed for the red grape must could be due to the commercial strain of *Saccharomyces cerevisea* (Fermol^®^ Grand Rouge from AEB, Viseu, Portugal) used. With respect to OAA, the concentrations were similar (*p* > 0.05) over the fermentation process, except in the first point “before fermentation” for the red grape must sample (R-BF), where it was only possible to detect traces of OAA. This lower value at the beginning of the fermentation process could be due to a lower extent of maceration observed in this point of the fermentation. Furthermore, the levels of OAA in white grape musts were slightly higher (~13 mg/L) compared to the levels observed in red grape musts (~6 mg/L) (Table 2). This could be correlated with the presence of anthocyanins observed in red grape musts and the possibility of reaction of the OAA with these compounds, as described previously herein.

**Figure 7:**
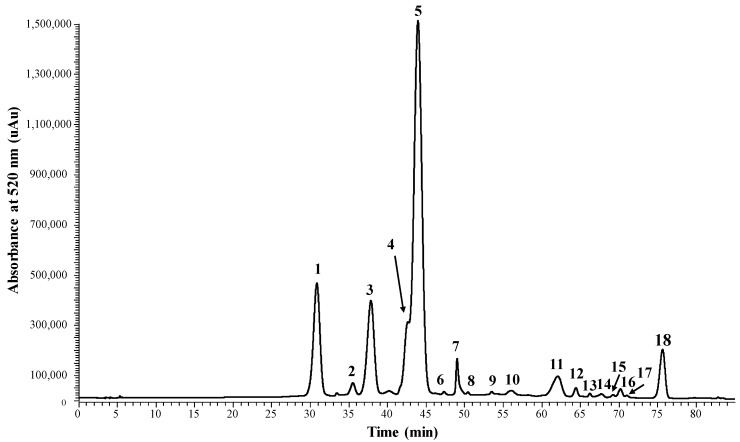
LC chromatogram recorded at 520 nm of the red grape must sample collected at the end of the alcoholic fermentation process (R-EF). 1, Dp-3-glc; 2, Cy-3-glc; 3, Pt-3-glc; 4, Pn-3-glc; 5, Mv-3-glc; 6, CarboxypyranoPn-3-glc; 7, CarboxypyranoMv-3-glc; 8, PyranoMv-3-glc; 9, CarboxypyranoMv-3-acetylglc; 10, Pt-3-acetylglc; 11, Mv-3-acetylglc; 12, Dp-3-coumglc; 13, CarboxypyranoMv-3-coumglc; 14, Mv-3-cafeyolglc; 15, Cy-3-coumglc; 16, Pt-3-coumglc; 17, Mv-3-*cis*-coumglc; 18, Mv-3-*trans*-coumglc.

In fact, four A-type vitisins (carboxypyranopn-3-glc, carboxypyranomv-3-glc, carboxypyranomv-3-acetylglc, and carboxypyranomv-3-coumglc) were found to occur in the red grape must sample collected at the end of the fermentation (R-EF) using LC-MS (Figure 7). The mv-3-glc A-type vitisin was also detected by LC-MS in the earliest stages of the must fermentation [24].

## 3. Materials and Methods

### 3.1. Reagents

Acetonitrile (HPLC grade), formic acid (99–100%), and methanol (p.a.) were purchased from Chem-Lab (Zedelgem, Belgium). Pyruvic acid (≥97%), oxaloacetic acid (≥97%), and PFBHA (*O*-(2,3,4,5,6-pentafluorobenzyl)hydroxylamine hydrochloride) (≥98%) were obtained from Sigma Aldrich (Madrid, Spain). LiChroprep^®^ RP-18 (40–63 µm) gel was obtained from Merck (Darmstadt, Germany).

### 3.2. Grape Must Samples

The red grape must samples were collected at different points of the fermentation process (before, middle, end) from a local winery from the Vinhos Verdes Region pomace maceration (c.v. Vinhão) in the harvest of 2016. The first sample (before fermentation) was collected after the grapes crushing and before the fermentation started (180 g/L of sugar). Then, the grape must was inoculated with *Saccharomyces cerevisea* (Fermol^®^ Grand Rouge, Pascal Biotech from AEB, Viseu, Portugal) and the next sample (middle of fermentation) was collected when half (109 g/L of sugar) of the initial sugar was converted into ethanol. The last sample was collected when the amount of residual sugar was 2 g/L.

The white grape must samples were obtained from Proenol—Indústria Biotecnológica SA. The white grapes (mixture of varieties) from the Vinhos Verdes Region (harvest of 2016) were pressed and then the white must was filtered using a 0.45 µm membrane. The must was then inoculated with *Saccharomyces cerevisea* (QA23^®^ from Proenol, V. N. Gaia, Portugal). The first sample (before fermentation) was collected after the inoculation. The second sample (middle of fermentation) was obtained when half (95 g/L of sugar) of the initial sugar was converted into ethanol. The last sample (end of fermentation) was collected when the sugar concentration was below 2 g/L (0.07 g/L).

All the samples were frozen (−20 °C) until use.

### 3.3. Malvidin-3-O-glucoside (Mv-3-glc)

A fraction containing mv-3-glc was obtained by fractionation of a young red wine extract in a Büchner funnel system with a porous plaque (porosity 3) under vacuum using RP-18 gel as stationary phase and 20% (*v*/*v*) aqueous methanol acidified with HCl as mobile phase. Mv-3-glc was further isolated from the obtained fraction by low pressure RP-18 gel column chromatography eluting with an aqueous solution of 20% (*v*/*v*) methanol acidified with HCl. The purity of the mv-3-glc was checked by HPLC-DAD.

### 3.4. Reactivity of Mv-3-glc with PA and OAA

Mv-3-glc (1.9 mM) was incubated with PA or OAA at different molar ratios (1:0.5, 1:1, 1:5, 1:10, 1:50, and 1:100) in 5 mL of water, at pH 2.6. The reactivity of mv-3-glc with PA and OAA was also tested at pH 1.5 and 3.5 using the solution with anthocyanin:organic acid molar ratio of 1:10. All reactions were kept at room temperature and the formation of the A-type vitisin was monitored by HPLC-DAD over 7 days.

To evaluate the preference of mv-3-glc to the organic acids (OAA or PA), a solution containing mv-3-glc (1.9 mM), OAA (9.5 mM), and PA (9.5 mM) was incubated at wine pH (pH = 3.5). The decrease of the OAA and PA content with time and the formation of the A-type vitisin were evaluated by HPLC-DAD.

### 3.5. High Performance Liquid Chromatography (HPLC–DAD) Analysis

All samples were analyzed using a Thermo^®^ Scientific HPLC with a Thermo^®^ Scientific Spectra System P4000 pump on a 250 × 4.6 mm i.d. reversed-phase C18 column (Merck^®^, Darmstadt, Germany). Detection was carried out between 200–800 nm using a diode array detector (Thermo Scientific Spectra System, Waltham, MA, USA, UV8000). Twenty microliters of each sample were injected using an autosampler Thermo^®^ Scientific Spectra System AS3000, as described in the literature by Oliveira et al. (2013) [25]. Solvents were: A, HCOOH/H_2_O (1:9, *v*/*v*) and B, HCOOH/CH_3_CN/H_2_O (1:3:6, *v*/*v*/*v*). The gradient consisted of 80–45% A for 38 min, at a flow rate of 1.0 mL/min. The column was washed with 100% B for 10 min and then stabilized at the initial conditions for another 10 min. The concentration of A-type vitisin (mg/L) and respective yields of reactions were determined using a calibration curve obtained for A-type vitisin, at different concentrations.

The detection and quantification of OAA and PA in model solutions was performed in a Thermo^®^ Scientific HPLC by injecting 20 µL of each sample on a 300 × 7.8 mm i.d. anion exclusion column (Grace Davison, Columbia, SC, USA) at 50 °C. The detection was carried out at 214 nm and the solvent used was 2.5 mM of H_2_SO_4_ at 0.35 mL/min for 30 min. Calibration curves were obtained using OAA and PA standards.

### 3.6. LC-MS Analysis

Grape must samples and the end product of the reaction of mv-3-glc with OAA were analyzed by LC-MS on a *Finnigan Surveyor* series liquid chromatograph, equipped with a 150 × 4.6 mm i.d., 5 µms LicroCART^®^ (Darmstadt , Germany) reversed-phase C18 column thermostatted at 25 °C. The mass detection was carried out by a Finnigan LCQ DECA XP MAX (Finnigan Corp., San José, CA, USA) mass detector with an API (Atmospheric Pressure Ionization) source of ionization and an ESI (ElectroSpray Ionization) interface. Solvents were A: H_2_O/HCOOH (99:1), and B: HCOOH/H_2_O/CH_3_CN (1:69:30). The HPLC gradient used was the same reported above for the HPLC analysis with the exception of the flow, which in this case was 0.5 mL/min. The capillary voltage was 4 V and the capillary temperature 300 °C. Spectra were recorded in positive ion mode between *m*/*z* 300 and 1500. The mass spectrometer was programmed to do a series of three scans: a full mass, a zoom scan of the most intense ion in the first scan, and a MS-MS of the most intense ion using relative collision energy of 30 and 60 V.

### 3.7. Statistical Analysis

The data analysis (triplicates) was performed by Analysis of Variance (ANOVA) using the GraphPad Prism 5 program (La Jolla, CA, USA). The differences between the means of the yields of the reactions were estimated using the Bonferroni test at *p* < 0.05.

## 4. Conclusions

Overall, the occurrence of A-type vitisins in red wines could be due to the reaction of mv-3-glc with PA, as already documented in the literature. In this work, overall, this data strongly supports the occurrence of a new chemical pathway that leads to the formation of A-type vitisins in red wine from the reaction between anthocyanins and OAA. This organic acid was shown to be more reactive with anthocyanins than PA, even using small molar ratios such as 1:05 and 1:1 that are more likely to occur in red wines. Although the concentrations of PA in red grape musts are much higher than the concentrations of OAA, the enhanced reactivity of anthocyanins with OAA at wine pH when compared to PA suggests that the A-type vitisin observed in red grape musts during fermentation should be formed from the reaction of anthocyanins with OAA and thereafter with PA during the ageing process.

## Figures and Tables

**Table 1:** Yields (%) of reaction for the synthesis of A-type vitisin from the reaction of mv-3-glc with oxaloacetic acid (OAA) or pyruvic acid (PA) at different pH values after 7 days of reaction at room temperature. Values with different letters are statistically different (*p* < 0.05).

Organic Acid	OAA			PA		
**pH**	**1.5**	**2.6**	**3.5**	**1.5**	**2.6**	**3.5**
**Yield (%)**	5.4 ± 0.6 ^a^	59 ± 3 ^b^	48 ± 3 ^c^	0.8 ± 0.1 ^a^	44 ± 2 ^c^	20.9 ± 0.5 ^d^

**Table 2:** Oxaloacetic (OAA) and pyruvic acid (PA) concentrations (mg/L) in white and red grape musts at different points of the grape must fermentation. Values with different letters are statistically different (*p* < 0.05).

Type of Must	Samples	Sugar Concentration (g/L)	OAA (mg/L)	PA (mg/L)
Red grape must samples	R-BF	180	<0.03 ^a^	46.40 ± 0.02 ^c^
	R-MF	109	5.5 ± 0.2 ^b^	243 ± 28 ^f^
	R-EF	2	6.1 ± 0.9 ^b^	250 ± 13 ^f^
White grape must samples	W-BF	213	14.0 ± 0.8 ^c^	224 ± 16 ^f,g^
	W-MF	95	11.4 ± 0.2 ^d^	320 ± 26 ^h^
	W-EF	0.07	13.0 ± 0.1 ^c^	163 ± 25g ^e,o^

R-BF, Red grape must sample—Before Fermentation; R-MF, Red grape must sample—Middle of Fermentation; R-EF, Red grape must sample—End of Fermentation; W-BF, White grape must sample—Before Fermentation; W-MF, White grape must sample—Middle of Fermentation; W-EF, White grape must sample—End of Fermentation.

## Supplementary Materials

Supplementary materials can be found at www.mdpi.com/1422-0067/18/4/762/s1.

## Abbreviations

PA	Pyruvic acid
OAA	Oxaloacetic acid
Mv-3-glc	Malvidin-3-*O*-glucoside
Dp-3-glc	Delphinidin-3-*O*-glucoside
Cy-3-glc	Cyanidin-3-*O*-glucoside
Pt-3-glc	Petunidin-3-*O*-glucoside
Pn-3-glc	Peonidin-3-*O*-glucoside
W-BF	White grape must—Before fermentation
W-MF	White grape must—Middle of fermentation
W-EF	White grape must—End of fermentation
R-BF	Red grape must—Before fermentation
R-MF	Red grape must—Middle of fermentation
R-EF	Red grape must—End of fermentation

## References

[1] Boss P.K., Davies C., Robinson S.P. (1996). Anthocyanin composition and anthocyanin pathway gene expression in grapevine sports differing in berry skin colour. Aust. J. Grape Wine Res..

[2] Morata A., Gómez-Cordovés M.C., Colomo B., Suárez J.A. (2003). Pyruvic acid and acetaldehyde production by different strains of *Saccharomyces cerevisiae*: Relationship with vitisin A and B formation in red wines. J. Agric. Food Chem..

[3] Bakker J., Timberlake C.F. (1997). Isolation, identification, and characterization of new color-stable anthocyanins occurring in some red wines. J. Agric. Food Chem..

[4] Gomez-Alonso S., Blanco-Vega D., Victoria Gomez M., Hermosin-Gutierrez I. (2012). Synthesis, isolation, structure elucidation, and color properties of 10-acetyl-pyranoanthocyanins. J. Agric. Food Chem..

[5] He J., Santos-Buelga C., Silva A.M.S., Mateus N., de Freitas V. (2006). Isolation and structural characterization of new anthocyanin-derived yellow pigments in aged red wines. J. Agric. Food Chem..

[6] Mateus N., Silva A.M.S., Vercauteren J., de Freitas V. (2001). Occurrence of anthocyanin-derived pigments in red wines. J. Agric. Food Chem..

[7] Pretorius I.S. (2000). Tailoring wine yeast for the new millennium: Novel approaches to the ancient art of winemaking. Yeast.

[8] Radler F., Fleet G.H. (1992). Yeasts—Metabolism of organic acids. Wine Microbiology and Biotechnology.

[9] Romero C., Bakker J. (2000). Effect of acetaldehyde and several acids on the formation of vitisin A in model wine anthocyanin and colour evolution. Int. J. Food Sci. Technol..

[10] Morata A., Calderon F., Gonzalez M.C., Gomez-Cordoves M.C., Suarez J.A. (2007). Formation of the highly stable pyranoanthocyanins (vitisins A and B) in red wines by the addition of pyruvic acid and acetaldehyde. Food Chem..

[11] Burns T.R., Osborne J.P. (2013). Impact of malolactic fermentation on the color and color stability of pinot noir and merlot wine. Am. J. Enol. Vitic..

[12] Oliveira J., Mateus N., de Freitas V. (2013). Network of carboxypyranomalvidin-3-*O*-glucoside (vitisin A) equilibrium forms in aqueous solution. Tetrahedron Lett..

[13] Oliveira J., Fernandes V., Miranda C., Santos-Buelga C., Silva A., de Freitas V., Mateus N. (2006). Color properties of four cyanidin-pyruvic acid adducts. J. Agric. Food Chem..

[14] Mateus N., Oliveira J., Santos-Buelga C., Silva A.M.S., de Freitas V. (2004). NMR structure characterization of a new vinylpyranoanthocyanin-catechin pigment (a portisin). Tetrahedron Lett..

[15] Oliveira J., de Freitas V., Silva A.M.S., Mateus N. (2007). Reaction between hydroxycinnamic acids and anthocyanin-pyruvic acid adducts yielding new portisins. J. Agric. Food Chem..

[16] Oliveira J., Azevedo J., Silva A.M.S., Teixeira N., Cruz L., Mateus N., de Freitas V. (2010). Pyranoanthocyanin dimers: A new family of turquoise blue anthocyanin-derived pigments found in Port wine. J. Agric. Food Chem..

[17] He J., Oliveira J., Silva A.M.S., Mateus N., de Freitas V. (2010). Oxovitisins: A new class of neutral pyranone-anthocyanin derivatives in red wines. J. Agric. Food Chem..

[18] Ribereau-Gayon P., Glories Y., Maujean A., Dubourdieu D. (2006). Organic acids in wines. Handbook of Enology: The Chemistry of Wine and Stabilization and Treatments.

[19] Ribéreau-Gayon P., Dubourdieu D., Donèche B., Lonvaud A. (2006). The grape and its maturation. Handbook of Enology.

[20] Shapiro F., Silanikove N. (2011). Rapid and accurate determination of malate, citrate, pyruvate and oxaloacetate by enzymatic reactions coupled to formation of a fluorochromophore: Application in colorful juices and fermentable food (yogurt, wine) analysis. Food Chem..

[21] Noguchi K., Mizukoshi T., Miyano H., Yamada N. (2014). Development of a new LC-MS/MS method for the quantification of keto acids. Chromatography.

[22] De Freitas V., Mateus N. (2011). Formation of pyranoanthocyanins in red wines: A new and diverse class of anthocyanin derivatives. Anal. Bioanal. Chem..

[23] Asenstorfer R.E., Markides A.J., Iland P.G., Jones G.P. (2003). Formation of vitisin A during red wine fermentation and maturation. Aust. J. Grape Wine Res..

[24] Araújo P., Fernandes A., de Freitas V., Oliveira J. (2017). Detection of A-Type Vitisins in Red Grape Must at the Beggining of Fermentation.

[25] Oliveira J., da Silva M.A., Jorge Parola A., Mateus N., Brás N.F., Ramos M.J., de Freitas V. (2013). Structural characterization of a A-type linked trimeric anthocyanin derived pigment occurring in a young Port wine. Food Chem..

