# Supplementary material for: A New Chemical Pathway Yielding A-Type Vitisins in Red Wines
Source: Int J Mol Sci. 2017 Apr 4;18(4):762. doi: 10.3390/ijms18040762 (PMC5412346; doi:10.3390/ijms18040762)
Supplement: Supplementary file 1 [file ijms-18-00762-s001.pdf]

Supplementary Materials:A New Chemical Pathway Yielding A-Type Vitisins in Red Wines

Paula Araújo, Ana Fernandes, Victor de Freitas and Joana Oliveira

S1. Identification of the new compound formed during the reaction of mv-3-glc with OAA

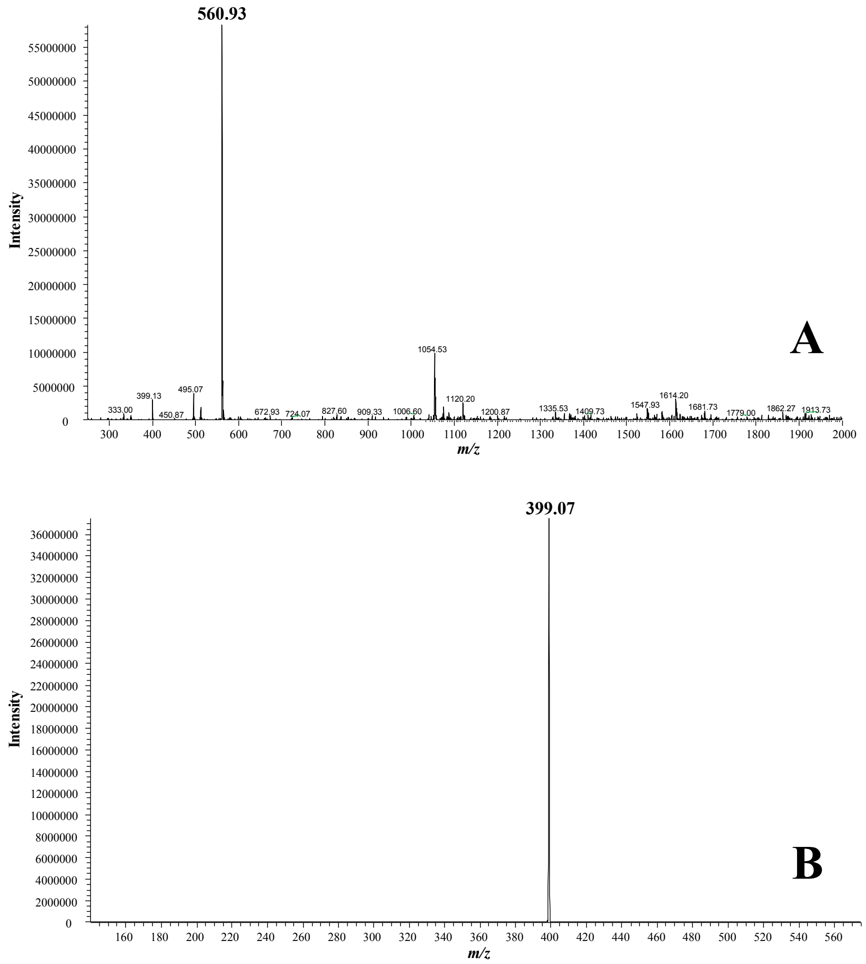

Figure S1. MS-ESI analysis performed in the positive-ion mode of the new compound formed during the reaction of mv-3-glc with OAA: (**A**) full mass spectrum; (**B**) MS^2 spectrum of the fragment obtained in full mass spectrum (*m/z* 561).

S2. Decomposition of oxaloacetic acid into pyruvic acid in model solution at pH 3.5

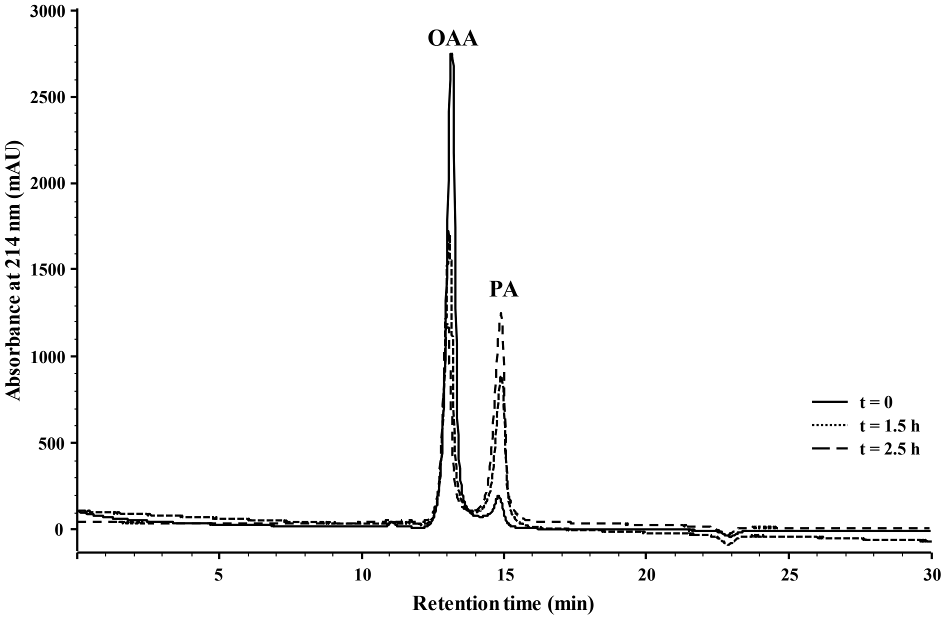

Figure S2. HPLC chromatograms recorded at 214 nm of a model solution (pH 3.5) containing OAA (7.6 mM) at pH 3.5 at time 0, 1.5, and 2.5 h at room temperature.

S3. Detection and quantification of OAA and PA in grape musts

3.1. Derivatization of Keto-Acids (OAA and PA)

The derivatization (oxime-derivatives) of the keto-acids OAA and PA was performed according to the procedure described in the literature by Noguchi and co-workers, 2014, with some changes [15].

Twenty microliters of the sample were mixed with 20 μL of PFBHA (*O*-(2,3,4,5,6-pentafluorobenzyl)hydroxylamine hydrochloride) 10 mg/mL in a microtube and allowed to react at 0 °C during 30 min (conversion of the keto-acids to the corresponding *O*-PFBO derivatives (*O*-(2,3,4,5,6-pentafluorobenzyl)oxime)). The derivatization reaction was quenched by the addition of 10 μL of a solution of acetone and acetonitrile (1:1, *v*/*v*), and then the resulting mixture was diluted with 25 μL of 0.1% NaOH and acetonitrile (1:1, *v*/*v*). This final mixture was then stored at 4 °C until LC-ESI-MS analysis. Standards of OAA and PA were prepared similarly as described for grape must samples. The samples and standards were analysed in triplicates.

3.2. LC/ESI-MS Analysis

Keto-acids *O*-(2,3,4,5,6-pentafluorobenzyl)oxime derivatives were analyzed by LC/ESI-MS on a Finnigan Surveyor series liquid chromatograph and their detection was done by mass spectrometry. The auto-sampler temperature and injection volume were set at 4 °C and 25 μL, respectively. Liquid chromatography was performed at 40 °C using a BDS Hypersil C18 column of (150 × 4.6 mm, 3 μm) and a gradient elution system with the mobile phase consisting of solvent A (50 μM formic acid in water) and solvent B (acetonitrile). The flow rate was maintained at 0.5 mL/min through the analysis. The gradient elution conditions consisted of 10–100% B for 30 min. The column was washed with 100% B for 6 min and then stabilised at the initial conditions for 5 min. The mass detector was a Finnigan LCQ DECA XP MAX (Finnigan Corp., San Jose, CA, USA) quadrupole ion trap equipped with atmospheric pressure ionization (API) source, using electrospray ionization (ESI) interface. The MS was operated in a Select Ion Monitoring (SIM) mode with negative detection. The vaporizer and the capillary voltages were 5 kV and 4 V, respectively. The capillary temperature was set at 325 °C. Nitrogen was used as both sheath and auxiliary gas at flow rates of 80 and 30, respectively (in arbitrary units).
